# Single Nucleotide Polymorphism Analysis of European Archaeological *M. leprae* DNA

**DOI:** 10.1371/journal.pone.0007547

**Published:** 2009-10-22

**Authors:** Claire L. Watson, Diana N. J. Lockwood

**Affiliations:** Department of Infectious Diseases, London School of Hygiene and Tropical Medicine, London, United Kingdom; New Mexico State University, United States of America

## Abstract

**Background:**

Leprosy was common in Europe eight to twelve centuries ago but molecular confirmation of this has been lacking. We have extracted *M. leprae* ancient DNA (aDNA) from medieval bones and single nucleotide polymorphism (SNP) typed the DNA, this provides insight into the pattern of leprosy transmission in Europe and may assist in the understanding of *M. leprae* evolution.

**Methods and Findings:**

Skeletons have been exhumed from 3 European countries (the United Kingdom, Denmark and Croatia) and are dated around the medieval period (476 to 1350 A.D.). we tested for the presence of 3 previously identified single nucleotide polymorphisms (SNPs) in 10 aDNA extractions. *M. leprae* aDNA was extracted from 6 of the 10 bone samples. SNP analysis of these 6 extractions were compared to previously analysed European SNP data using the same PCR assays and were found to be the same. Testing for the presence of SNPs in *M. leprae* DNA extracted from ancient bone samples is a novel approach to analysing European *M. leprae* DNA and the findings concur with the previously published data that European *M. leprae* strains fall in to one group (SNP group 3).

**Conclusions:**

These findings support the suggestion that the *M. leprae* genome is extremely stable and show that archaeological *M. leprae* DNA can be analysed to gain detailed information about the genotypic make-up of European leprosy, which may assist in the understanding of leprosy transmission worldwide.

## Introduction

Leprosy remains a public health problem with over 210,000 registered cases worldwide at the beginning of 2008. *Mycobacterium leprae (M. leprae)*, is an obligate intracellular parasite and has proved to be uncultivable on artificial medium only growing in susceptible animal models such as the foot pads of mice [1] and the nine banded armadillo [2].

Leprosy is thought to have been brought to Britain by Roman armies that had been based in Asia and the Middle East previously. The prevalence of leprosy increased in Europe after 1000 A. D. and increased up until the 14–15^th^ Century A. D. when a rapid decline was observed, the cause of this is unknown. Leprosy remained in Scandinavia until the 16^th^ century when it disappeared mostly, only remaining in Norway [3]. The last case of leprosy in Norway was registered in 1953 [4]. Today, the majority of European leprosy cases are considered to be imported from leprosy endemic countries [5].

The obligate intracellular status of *M. leprae* is probably due to the extreme reduction of the genome, at 3.3 Mb it has lost almost 2,000 genes in comparison to *Mycobacterium tuberculosis* [6]. Less than half of the *M. leprae* genome contains functional genes and gene deletion and decay appears to have eliminated many important metabolic activities, including part of the oxidative and most of the microaerophilic and anaerobic respiratory chains [7]. Clinical leprosy presents with a spectrum of features ranging from localised tuberculoid disease to widespread lepromatous disease. If left untreated, the mycobacterium can directly invade the skeleton of its host, giving rise to characteristic destructive leprous osteomyelitis lesions that can be identified long after the death of the individual [8]. Bone changes are most frequently identified in the hands and feet of leprosy patients, other lesions include localised osteoporosis, honeycombing and concentric bone absorption [9].

The principle method of pathogen DNA survival within an archaeological specimen is unknown. Very little is known about the levels of pathogen DNA preserved in bone and the ability of this pathogen to survive in bone following the death of the host. Most pathogens are at a particular disadvantage as they do not invade the bone structure and have a weak cell wall. *M. leprae* in comparison, is known to invade the macrophages of the host and has a thick, waxy, mycolic acid coating. It has been suggested that this component has a protective role, enhancing the survival of Mycobacterial DNA in archaeological samples [10], [11].

The first isolation of mycobacterial DNA from archaeological samples was by Spigelman *et al* in 1993 [12], who developed a technique using PCR amplification to identify degraded, genetic material in ancient bone samples. The publication detailed PCR protocols, bone preparation and the findings from several pilot studies, indicating how mycobacterial DNA might be extracted from ancient bone samples [12]. This technique was used to isolate, *M. tuberculosis* DNA from lung lesions (N1 and N2) of a spontaneously mummified, 1000-year-old adult female body in southern Peru, using the amplification of a 123 bp segment of the IS6110 element specific to *M. tuberculosis*. Following this work, several researchers published work on the presence of *M. tuberculosis* DNA in archaeological material including bone [13], calcified pleura [14] and mummified remains [15].

In 1994, *M. leprae* DNA was successfully isolated from ancient human bone samples over 1000 years old and PCR assay confirmed the presence of an *M. leprae* specific segment of DNA sequence (RLEP) [16]. Later, Haas *et al* [17] extracted *M. leprae* specific DNA fragments (RLEP1 and RLEP3) from skeletal remains exhumed from a South German ossuary and a Hungarian cemetery.

More recently, the analysis of *M. leprae* aDNA extracted from archaeological material became more detailed with the inclusion of variable nucleotide tandem repeat (VNTR) analysis [18]. Following this work, Monot *et al* [19] compared the stability of two different markers of genomic biodiversity of *M. leprae* in several biopsy samples isolated from the same leprosy patient (VNTRs and SNPs). The group observed no variation in the SNP profiles but considerable variation in the VNTR profiles, suggesting that VNTR analysis may be too dynamic for use as epidemiological markers for leprosy. The identification of SNPs in the modern *M. leprae* genome has only been completed recently [19]. It is thought that the identification of these SNPs in pathogenic bacteria may assist in the understanding of important factors, such as disease susceptibility, the location of real locus involved in disease development and the epidemiology of the bacteria [19].

The present report describes the molecular methods used to test for the presence of 3 previously published SNPs in *M. leprae* DNA extracted from ancient European skeletal remains. The methods, termed SNP typing include PCR amplification and sequencing of areas of the *M. leprae* genome known to contain SNPs of interest that have been previously described [19]. The PCR assays have been applied to aDNA extracted from skeletons exhumed in 3 European countries with the aim of comparing these findings to SNP data already available for world-wide modern *M. leprae* DNA.

## Materials and Methods

Ethical approval to work with ancient human material was obtained for all sample sites and also from the ethics committee at the London School of Hygiene and Tropical Medicine (LSHTM). For the UK samples, ethical approval was given by Cambridgeshire County Council, Archaeological Field Unit and the English Heritage, Centre for Archaeology, Portsmouth. For samples collected in Copenhagen, approval was granted by the Medical History Museum and in Odense, Denmark, approval was gained through Odense University ethics committee. For the remaining locations, ethical approval was granted during excavation by the governing body responsible for the skeletal material and burial site.

10 bone samples were collected from skeletons exhumed from the UK (Norwich) and Europe (Denmark and Croatia) (Table 1). Samples were selected at random from the European archaeological bone collection at LSHTM and all samples in the collection were taken from a site on the skeleton most likely to have been invaded by *M. leprae* including the rhino-maxillary area and hand and foot bones [20]. All the skeletons showed typical signs of leprous osteomylitis, including resorption of the anterior nasal spine, rounding and widening of the nasal aperture, erosion of the alveolar margin and pitting of the hard palate. The long bones showed deposits of woven bone and the hands and feet showed honeycombing and concentric bone absorption. Bone samples were stored at minus 20 degrees centigrade until analysis.

**Table 1:** Skeleton sample information, location, period and burial dates.

Country	Sample reference	Skeletal sample site	Burial Location	Period	Number of samples
**Croatia**	1A	Rhino-maxillary	Radasinovci	8^th^–9^th^ Century AD	4
	2A				
	3A				
	4A				
**Denmark**	G483	Palatine	Odense Leprosarium	1275–1560 AD	1
**UK**	11784	Rhino-maxillary	St. John's Timberhill, Norwich	900–1000 AD	5
	11287	5^th^ metatarsal			
	11503	Tibia lesion			
	11287	5^th^ metatarsal			
	11428	Rhino Max			

Skeletons used for teaching purposes or for museum displays, were sampled sparingly from the rhino-maxillary area or an area that would not damage the appearance of the bones. Skeletal samples were either sampled on site by the author, or a protocol was sent to the curator of the collection who would sample the material.

In some cases, curators preferred to sample the skeletons or access was not possible. For these cases, a protocol was designed to allow curators of bone collections to sample the material effectively and safely without the presence of the author. Briefly, this protocol included requesting the sampler to wear gloves for each sample, use a sterile scalpel blade and sample on a disposable surface such as paper towel or a clean sheet of A4 paper. The sampler was requested to collect around 100 mg (0.1 gram) of bone from an area most likely to have experienced direct bacterial invasion including the rhino-maxillary area, hands, feet and nasal bones. The long bones (Leg bones) of the skeleton are also a possible source where typical lepromatous changes had occurred. Each sample was stored in a sterile tube and transported back to the lab with the related log sheet providing as much information as possible about the samples.

Once the samples had been collected, they were placed inside a padded envelope and transported to the London School of Hygiene and Tropical Medicine via the postal service or a courier selected by the curator. The samples were kept as cool as possible during the journey.

Each skeleton was examined paleopathologically by the curator of the collection and the sex, approximate age, burial date and excavation location were recorded along with any skeletal lesions. The sex of the skeleton was ascertained using pelvic and skull comparison measurement techniques. Radio carbon dating was carried out where possible, when not possible, the burial date was estimated from other artefacts buried with the skeletons and the location and position of the site. A log sheet was filled in for each sample taken and the curator was requested to provide any literature available about the burial site and skeletons sampled.

The bones included in this study have been stored in separate, sealed containers following extraction and were cleaned by the related institutions following their own protocols. All the reagents are specifically dedicated to aDNA extraction and are stored separately from other extraction reagents in the lab. Protective sterile gloves were worn during the extraction, which was carried out on a dedicated bench using sterile tools, tubes and disposable bench coat (changes after every extraction. The bench was cleaned with DNAse away (Molecular Bioproduct, San Diego, CA) before and after every aDNA extraction and reusable equipment such as the pestle and mortars were autoclaved before use and lab coats were clean. Since we were not working with human DNA, the use of protective clothing such as masks and booties was considered unnecessary. Only 1 set of aDNA extractions were completed each week and the lab was cleaned thoroughly before commencing the next extraction. At the time of aDNA extraction, the laboratory worked only with aDNA and one modern Indian *M. leprae* isolate. In the laboratory, samples were weighed and then ground down to a fine powder in a sterile pestle and mortar on a clean bench. A modified version of the protocol used by Bouwman and Brown [21] was used for aDNA extraction. Briefly, the bone powder was placed into a 15 ml tube and 1 ml of extraction buffer (0.5 M EDTA pH 8, 0.5% Tris, 100 µg/ml-1 proteinase K) (Nuclisens, Biomerieux) was added to each tube including an empty tube to be used as an extraction blank control. The tube was incubated at 55°C for 24 hours and then underwent three freeze-thaw cycles in liquid nitrogen. Once fully defrosted the samples were centrifuged at 2000 rpm for 5 minutes. The supernatant was transferred to a new 15 ml tube containing 2.5 ml PB buffer (QIAGEN, West Sussex) and mixed gently. 0.75 ml of this solution was added onto a QIAquick column and centrifuged at 14,000 rpm for 1 min. The buffer collected in the external tube was discarded. This step was repeated until all the solution had been passed through the column. 0.75 ml PE buffer (QIAGEN, West Sussex) was added to each column. Following centrifugation at 14,000 rpm for 1 minute, the buffer was discarded and the internal section of the column was transferred into clean tubes. 50 µl of extraction buffer (QIAGEN, West Sussex) was added to the centre of each column and incubated for 1 minute. The columns were centrifuged at 14,000 rpm for 1 minute and the eluate collected in a fresh 0.5 ml non-stick Eppendorf tube. The extract was stored at −80°C until analysis. Independent confirmation was provided by a second member of the laboratory in a different laboratory in a separate area of the institution. aDNA was extracted from bone sample G483 (Denmark) and amplified by to confirm result. The precious nature of the bone meant that duplication could not be carried out on all of the archaeological specimens.

The 3 previously published SNP assays [19] were used to identify SNPs in the ancient DNA extractions. However, any DNA persisting in old archival material is more likely to be damaged than that from more modern samples so PCR primers were designed to amplify smaller amplicons. All primers were calculated using the published *M. leprae* sequence [22] and checked using a Basic Local Alignment search tool (http://www.ncbi.nlm.nih.gov/BLAST/). Each primer was designed to be around 18–27 bp in length, have a Tm of around 64–70°C and have a G+C content lower than 50%. The RLEP (repetitive element) multi-copy target, specific to *M. leprae*, was used for initial screening of samples, to determine the presence of *M. leprae* DNA (Table 2). Primers for this were designed from the published RLEP primers [23]. SNP Primers can be viewed in Table 3 along with key PCR conditions and product sizes.

**Table 2:** RLEP primer sequences, melting temperatures, binding positions on DNA and NCBI accession numbers.

Primer	Sequence^a^	T_m_°C	Accession number	Amplicon size
RLEP F2	F 5′-CATTTCTGCCGCTGGTAT-3′	56.9	AL583917.1	111 bp
RLEP R4	R 5′-ATCATCGATGCACTGTTCAC-3′	56.6		

a F, forward; R, reverse.

**Table 3:** SNP primer sequences, melting points (T_m_), binding positions on DNA and NCBI accession numbers.

Primer set name	Primer Sequence	T_m_ °C	SNP location on genome	NCBI Accession number	Product size (bp)
SNP14676	F2 5′-ACGAATTCGTTGAACAGTCTC-3′	59.47	14676	AL583917.1	131
	R 5′-CAATGCATGCTAGCCTTAATGATAAA-3′	60.09			
SNP2935685	F2 5′-CTCGGAGAATTTCTATGCAAGTTTGA-3′	61.66	2935685	AL583925.1	151
	R 5′-ACCGGTGAGCGCACTAAG-3′	62.95			
SNP1642875	F2 5′-GGCTCGTCACAAATCCGAGTTT-3′	63.4	1642875	AL583921.1	115
	R 5′-GTAGTAGTCTTCCAAGTTGTGGTG-3′	63.69			

The DNA standard used as a positive control in the study consisted of 5 µg of DNA. This DNA was a modern isolate passaged through the armadillo, strain 4089, batch NAG 8.4.03C, supplied by Patrick Brennan through the NIH leprosy contract (http://www.cvmbs.consolate.edu/mip/leprosy/index.html). 5 µl of the stock solution was diluted in 100 µl of nuclease free water (Qiagen) and aliquoted into 5 tubes, each containing 21 µl of the diluted stock solution. The positive control DNA cannot be distinguished from the European aDNA when amplified using the RLEP primers used in the initial analysis, however, the control *M. leprae* DNA is most similar to the Tamil Nadu published sequence of *M. leprae* (it is a modern Indian isolate) and, therefore, belongs to group 4, showing a difference at all of the 3 SNP points used in the study, detecting contamination. The positive controls were run separately from the samples to reduce the risk of DNA transfer within the PCR machine and the DNA was only removed from the machine and added to the gel after all samples had been loaded.

All PCR assays included 2 negative controls which contained the PCR reaction mix and Nuclease free water (Qiagen). These were loaded before and after the *M. leprae* standard during the PCR amplification and electrophoresis to avoid cross-contamination via the tube or well. Cycling conditions for both the standards and the negative controls were kept the same as the sample conditions and the annealing temperature was determined by optimisation of the primer set.

All PCR reagents used in the study were dedicated for aDNA analysis and stored separately from other reagents. All PCR set up was carried out in a PCR workbench and UV light was used to decontaminate the area before and after any work. PCR assays were performed using the GeneAmp ® 2700 PCR system (Applied Biosystems, Cheshire, UK). The Horizon 11.14 tank (Invitrogen, Paisley, Scotland) was used for gel electrophoresis to check purity and DNA concentration of PCR products. PCR products were purified using the QIAquick PCR Purification Kit (Qiagen) according to manufacturer's instructions. 5 volumes of PB buffer (supplied in kit) were added to 1 volume of the PCR product and the solution added to a QIAquick spin column in a 2 ml collection tube. The column was centrifuged for 60 seconds to bind the DNA. The supernatant was discarded and the column washed in 0.75 ml PE buffer. To elute the DNA, 20 µl EB buffer was added to the centre of each column and incubated for 1 minute. The column was then centrifuged for 1 minute and the supernatant was collected. Cycle sequencing of the purified PCR products was carried out using the ABI BigDye v3.0 Cycle Sequencing Ready Reaction Kit (Applied Biosystems, Cheshire, UK) according to the manufacturers recommendations and the GeneAmp ® 2700 PCR system (Applied Biosystems, Cheshire, UK). Cycling conditions differed to the manufacturer's instructions and were as follows: 96°C for 30 seconds and 25 cycles of 96°C for 30 seconds, 50°C for 15 seconds and 60°C for 4 minutes.

Following cycle sequencing, the reactions were passed through the DyeEx 2.0 spin column kit (Qiagen) for dye terminator removal according to the manufacturer's instructions. Briefly, the product was loaded into the centre of the spin column and centrifuged for 3 minutes at low speed. The supernatant was then collected in a 1.5 ml no stick Micro tube (Alpha laboratories, Hampshire, UK) and freeze dried. Following re-suspension in formamide, the samples were analysed using the ABI3730 genetic analyzer (Applied Biosystems). Sequences were imported and analysed in the ABI sequence scanner V1.0 software downloaded from the Applied Biosystems website (http://www.appliedbiosystems.com/). All sequences were compared to those held in the NCBI database (http://www.ncbi.nlm.gov) using BLAST (Basic Local Alignment Search Tool) to find regions of local similarity.

## Results

**Figure 1:**
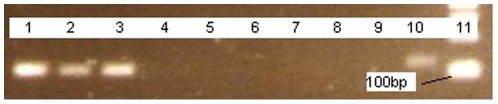
3% agarose gel showing clear bands for the aDNA extracts taken from sample G483, 2A and 3A, matching the size of the *M. leprae* positive control DNA following amplification with RLEP primers 2 & 4 (111 bp), Croatian samples 1A and 4A did not show a matching band and were not analysed further. *Key: 1. M. lep pos control DNA. 2. 2A (Croatia). 3. 3A (Croatia). 4. 4A (Croatia). 5. 1A (Croatia). 6. Extraction blank 1. 7. Extraction blank 2. 8. Water control 1. 9. Water control 2 (run with positive control). 10. G483 (Denmark). 11. 100 bp ladder.* N.B. The *M. leprae* positive control DNA (lane 1) was amplified separately from the extractions to avoid contamination.

The aDNA was confirmed as being from *M. leprae* by identifying the RLEP repetitive sequence using specially designed primers (primers 2 & 4) amplifying a 111 bp product. The RLEP repetitive sequence was detected in 3 samples from the United Kingdom (11784, 11287 and 11503), one sample from Denmark (G483) and 2 samples from Croatia (2A and 3A). This aDNA was sequenced in duplicate, using the QIAGEN Dye-ex column system and checked by BLAST search. Figure 1 shows an example of the electrophoresis gel. The remaining 4 DNA extractions (1A, 4A, 11287 and 11428) were negative for *M. leprae* DNA and were not analysed further.

**Figure 2:**
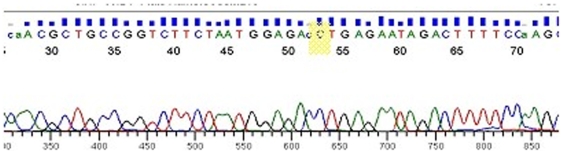
Sequence of SNP 14676 showing a “C” (highlighted in yellow) for aDNA extracted from St John's Timber Hill skeletal sample 11784 (rhino-max).

On SNP typing, a ‘C’ was found at SNP 14676 in *M. leprae* aDNA extracted from 6 DNA extractions from bone exhumed in the UK, Croatia and Denmark that contained the RLEP sequence. PCR amplicons were visualised on 3% agarose and a band of expected 131 bp size was seen following electrophoresis, the amplicons were then sequenced. All sequence data was confirmed using BLAST search and the results were duplicated to ensure accuracy. Figure 2 shows an example of the sequence data for SNP 14676. The sequence was also checked for differences in positions other than at the point of SNP mutation, no differences were observed.

**Figure 3:**
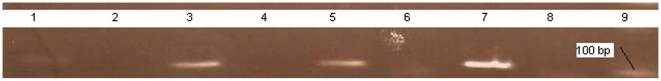
3% agarose gel stained with ethidium bromide visualising the reproduction of amplification of aDNA extraction from skeletal UK samples 11784, 11287 and 11503, following amplification with SNP-2935685 (151 bp). 1. Sample 11784. 2. Blank well. 3. Sample 11287. 4. Blank well. 5. Sample 11503. 6. Extraction blank. 7. *M. leprae* control DNA. 8. Negative water control. 9. 100 bp ladder.

**Figure 4:**
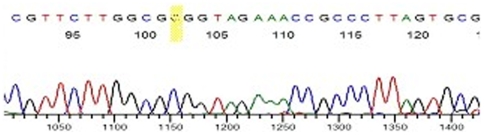
Sequence of SNP2935685 showing a “C” (highlighted in yellow) for aDNA extracted from Croatian skeletal sample 2A (rhino-max).

On SNP typing, a ‘C’ was found at SNP 2935685 in the *M. leprae* aDNA extracted from the 6 skeletal remains samples included in the SNP identification from the UK, Croatia and Denmark. The aDNA extractions were amplified by PCR using the SNP2935685 assay. Visualisation on 3% agarose confirmed a positive result and following sequencing (Figure 3), the data was checked using BLAST search and duplicated for accuracy. Figure 4 shows an example of the sequence data for SNP 2935685.

On SNP typing, a ‘T’ was found at SNP location 1642875 in the genome of aDNA extracted from all 6 skeletal remains samples from the UK, Croatia and Denmark. The aDNA was amplified by PCR using the SNP1642875 assay. Visualisation on 3% agarose confirmed a positive result and following sequencing, the data was checked using BLAST search and duplicated for accuracy.

Previously, the 3 SNP PCR assays (SNP14676, SNP1642875 and SNP2935685), were used to group 175 modern *M. leprae* isolates from 21 countries into 4 SNP types [19]. Europe was included in this study and consisted of 2 *M. leprae* isolates of French origin. Using this SNP typing method, The 6 aDNA isolates included in this study fall into SNP-type 3.

## Discussion

Evolutionary analysis of bacteria to address questions of biogeography are really limited and have mostly been done relatively recently. This study provides European archaeological *M. leprae* SNP data and is a novel approach to analysing the European *M. leprae* genome. We have isolated and analysed *M. leprae* DNA from 6 skeletons that are eight to twelve centuries old and obtained from widely separated geographical locations in Europe (UK, Denmark and Croatia). Previous molecular analysis of ancient *M. leprae* DNA has not included SNP analysis and European samples included in the previously published SNP research [19] relied on 2 undated biopsy samples, thought to be under 100 years old and of unclear provenance. Analysis of the *M. leprae* aDNA genome using SNP identification, included in this project, has provided a unique and insightful way of analysing skeletal remains.

Monot *et al* [19] published work identifying 3 SNP locations in the modern *M. leprae* genome that could be used to identify 4 SNP types, with the two *M. leprae* strains from France being SNP type 3. The data of this paper indicates that *M. leprae* has an extremely stable genome and that SNPs can be identified in modern clinical material that begin to provide a map of leprosy transmission worldwide. The decline of leprosy cases in European countries means that no modern material can be sourced for this SNP identification technique with any certainty that the strain is of European origin. SNPs in *M. leprae* aDNA extracted from skeletal remains from 3 European countries (Denmark, Croatia and the United Kingdom) have been successfully identified in this study, with the findings indicating that European leprosy isolates form SNP-type 3, as was previously suggested [19].

The findings of Monot *et al* [19], suggest that all cases of leprosy could be attributed to a single clone, with the dissemination of this clone being traceable, using SNPs to suggest that leprosy originated in Africa and spread by human migration. The group showed that the *M. leprae* strain responsible for leprosy in the European and North African countries was most similar to the strain responsible for most disease in the Americas and suggested that colonialism and emigration from the old world may have contributed to the introduction of leprosy into the new world. The findings from this study agree with the previously suggested transmission pattern and have worked towards being able to provide European SNP results in addition to the world-wide findings of the transmission map created at the Institut Pasteur [19].

The presence of *M. leprae* DNA in long bones, hands and feet of a skeleton is unusual but not overly surprising. Research into *M. tuberculosis* DNA analysis in archaeological specimens has shown that whilst the aDNA comes from a skeleton showing typical lesions, the sample of bone did not need to come from an area close to a lesion [24], [25], implying that the location of sampling may not be critical as the pathogen DNA was present in the blood stream, a theory proposed by Barnes and Thomas [26]. Periostitis with subperiosteal new bone deposits is not uncommon in the long bones of lepromatous leprosy patients [27]. Although it is not clear if this long bone damage is always due to direct *M. leprae* invasion, it is known that wherever the *M. leprae* are deposited by the macrophage, the bacilli colonise the locality, grow and produce lesions. A study on *M. lepraemurium* infected mice found that 3–5% of the mice showed bilateral paralysis of the rear limbs. Following dissection and within the bones the bone marrow was replaced by extended bacilli-laden granulomas that frequently eroded the bone wall [28].

Contamination of aDNA extractions is a major concern in this type of work and (especially for human aDNA extraction contaminated with modern human DNA) is a common problem for ancient DNA analysis. The rigorous methodology of Cooper & Poinar [29] to avoid modern or ancient DNA contamination must be considered and adhered to as much as possible, however, when looking at bacterial aDNA, one must put the environmental situation into context. aDNA extraction for the purpose of this study was carried out in a laboratory that has worked with modern Indian *M. leprae* DNA in the past and uses the DNA of a modern Indian *M. leprae* isolate as a positive control. The laboratory never receives European *M. leprae* DNA that could contaminate the ancient samples and the lack of genuine *M. leprae* isolates originating in European countries currently would make this a difficult process. The results were duplicated in a laboratory that does not work with *M. leprae* DNA, strengthening the probability that the results did not stem from contamination and although duplication of results in a dedicated human aDNA laboratory would further improve this probability, it should not be considered fundamental.
