# Correction: Single Nucleotide Polymorphism Analysis of European Archaeological *M. leprae* DNA

**DOI:** 10.1371/annotation/1b400b6e-8883-436c-b3c4-00e1ec2db101

**Published:** 2010-01-22

**Authors:** Claire L. Watson, Elizabeth Popescu, Jesper Boldsen, Mario Slaus, Diana N. J. Lockwood

Three authors were omitted from the author list. The corrected author list should read: Claire L. Watson, Elizabeth Popescu, Jesper Boldsen, Mario Slaus, Diana N. J. Lockwood. The citation should be: Watson CL, Popescu E, Boldsen J, Slaus M, Lockwood DNJ (2009) Single Nucleotide Polymorphism Analysis of European Archaeological *M. leprae* DNA. PLoS ONE 4(10): e7547. doi:10.1371/journal.pone.0007547

